# Recent Advancements in Smart Hydrogel-Based Materials in Cartilage Tissue Engineering

**DOI:** 10.3390/ma18112576

**Published:** 2025-05-31

**Authors:** Jakob Naranđa, Matej Bračič, Uroš Maver, Teodor Trojner

**Affiliations:** 1 Department of Orthopaedics, University Medical Centre Maribor, SI-2000 Maribor, Slovenia; jakob.naranda@gmail.com; 2 Institute of Biomedical Sciences, Faculty of Medicine, University of Maribor, SI-2000 Maribor, Slovenia; 3 Faculty of Mechanical Engineering, University of Maribor, SI-2000 Maribor, Slovenia; matej.bracic@um.si

**Keywords:** smart materials, cartilage tissue engineering, responsive hydrogels, biomaterials, omics-based approach

## Abstract

Cartilage tissue engineering (CTE) is an advancing field focused on developing biomimetic scaffolds to overcome cartilage’s inherently limited self-repair capacity. Smart hydrogels (SHs) have gained prominence among the various scaffold materials due to their ability to modulate cellular behavior through tunable mechanical and biochemical properties. These hydrogels respond dynamically to external stimuli, offering precise control over biological processes and facilitating targeted tissue regeneration. Recent advances in fabrication technologies have enabled the design of SHs with sophisticated architecture, improved mechanical strength, and enhanced biointegration. Key features such as injectability, controlled biodegradability, and stimulus-dependent release of biomolecules make them particularly suitable for regenerative applications. The incorporation of nanoparticles further improves mechanical performance and delivery capability. In addition, shape memory and self-healing properties contribute to the scaffolds’ resilience and adaptability in dynamic physiological environments. An emerging innovation in this area is integrating artificial intelligence (AI) and omics-based approaches that enable high-resolution profiling of cellular responses to engineered hydrogels. These data-driven tools support the rational design and optimization of hydrogel systems and allow the development of more effective and personalized scaffolds. The convergence of smart hydrogel technologies with omics insights represents a transformative step in regenerative medicine and offers promising strategies for restoring cartilage function.

## 1. Introduction

Cartilage is a load-bearing, tension-resistant tissue with limited ability for intrinsic self-repair. Cartilage tissue engineering (CTE) is a multidisciplinary field that merges biology, materials science, and engineering principles for cartilage regeneration and replacement [1]. Approaches like scaffolds, cell-seeded constructs, and bioprinting are being explored to develop tissue substitutes that replicate the structure and function of native cartilage [2]. The basic principles of CTE combine stem cells and engineered 3D biodegradable scaffolds, suitably functionalized with bioactive molecules, to promote cartilage tissue repair and regeneration [3]. Smart materials have become a groundbreaking tool in CTE, providing dynamic and reactive properties that improve the effectiveness of regenerative treatments. These materials can react to external factors like temperature, mechanical stress, pH, and biochemical signals, allowing them to be highly versatile in the complex environment of cartilage regeneration [4,5].

Smart materials are defined as materials capable of altering their functional or physical properties in response to environmental changes or external stimuli [6,7]. These materials, often referred to as intelligent or responsive materials, are widely used across various fields, including automotive, aerospace, and biomedical sectors. Developing bioresponsive scaffolds that can guide cellular behavior, promote extracellular matrix (ECM) deposition, and enhance tissue integration is crucial in tissue engineering [8,9,10]. While smart materials offer significant advantages, scalability, biocompatibility, and long-term stability must be addressed before widespread clinical application. Ongoing research is focused on optimizing these materials for better performance and ensuring their safety and efficacy in clinical settings. These materials are broadly categorized as active and passive [11,12]. Due to their unique ability to convert energy and alter their properties in response to specific stimuli, smart materials are ideal for use in tissue engineering [13].

**Figure 1:**
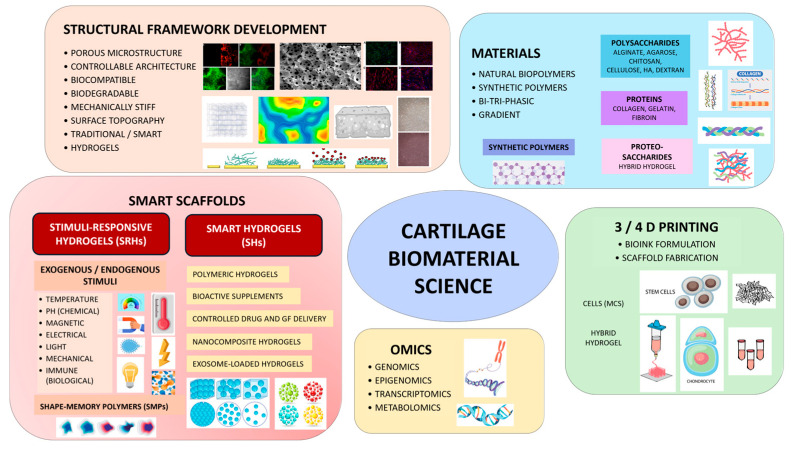
Schematic representations of fundamental techniques for scaffold formation and the application of smart materials in CTE.

In CTE, active smart materials like piezoelectric materials, polymeric hydrogels, and shape-memory polymers are particularly interesting due to their ability to interact dynamically with the tissue environment [5]. Incorporating nanotechnology further expands these materials’ capabilities, allowing for the design of nanoscale cues that direct stem cell differentiation and tissue formation [14,15]. Hydrogels, self-healing biomaterials, and shape-memory polymers are among the most promising smart materials being explored [16,17]. Hydrogels provide a hydrated environment that supports cell growth and can be engineered to release bioactive molecules in a controlled manner. Shape-memory polymers can alter their configuration to mimic the mechanical behavior of native cartilage, improving scaffold integration and durability. Additionally, self-healing biomaterials can potentially prolong the lifespan of cartilage implants by repairing minor damage autonomously [18,19,20]. Appropriate incorporation of bioactive molecules can be implemented to functionalize the hydrogel scaffolds. Previous studies have utilized various bioactive molecules, including chemokines and growth factors, to enhance the recruitment of bone mesenchymal stem cells (BMSCs) onto cartilage scaffolds [6]. Figure 1 presents a schematic illustration of fundamental scaffold formation techniques and smart materials’ application in CTE.

This review highlights the pivotal role of material selection in CTE and underscores the transformative potential of smart materials in developing responsive and adaptive scaffolds for improved tissue regeneration. Advanced fabrication methods are discussed. Sophisticated features, such as the self-healing ability of smart hydrogels (SHs) and the delivery of drugs and growth factors, are also highlighted. Nonetheless, a promising omics-based approach for fabricating SHs is underlined and suggested to guide future material selection.

## 2. Keyword Analysis

A thorough literature review was performed using major medical databases, including ScienceDirect, PubMed, and Springer Nature, to identify studies focused on smart materials and cartilage tissue engineering. The following specific keywords were used: “cartilage tissue engineering”, “smart materials”, “responsive hydrogels”, “biomaterials”, and “omics-based approach”. Additionally, the following Medical Subject Headings (MeSH) were utilized: “regenerative medicine”, “tissue engineering”, “articular cartilage”, “tissue scaffolds”, “cell differentiation”, “biocompatible materials”, “stimuli-responsive polymers”, “hydrogels”, “3D printing”, and “smart materials”. We identified recent high-impact studies on using smart scaffolds in CTE by applying this search algorithm along with specific filters (limited to the past five years and review articles).

**Figure 2:**
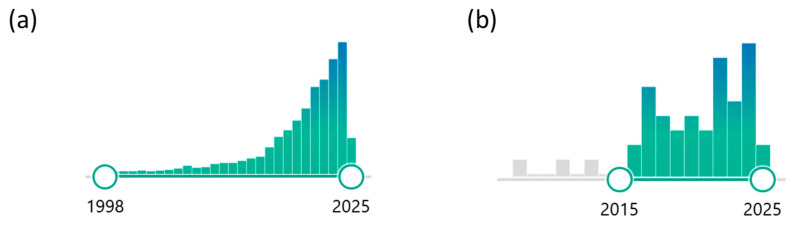
Several publications in PubMed (search conducted in March 2025) were identified using the following keywords: (**a**) “smart hydrogels” and (**b**) “smart hydrogel, cartilage tissue engineering”.

The growing number of publications on smart scaffolds highlights the increasing interest in this field. Specifically, over 2900 publications in PubMed on “smart hydrogels” demonstrate that this is an area of significant research activity (Figure 2a). However, when refining the search to include both “smart hydrogels” and “cartilage tissue engineering”, only 43 publications were found (Figure 2b), suggesting that while smart hydrogels are widely studied, their application in CTE remains relatively underexplored, presenting opportunities for further research and development.

## 3. Scaffolds in Cartilage Tissue Engineering

The main objective of tissue engineering is to regenerate a cellular construct capable of supporting the proliferation and differentiation of cells into a specific tissue type. Significant challenges arise in CTE due to the distinctive characteristics of cartilage, including specialized ECM composition, remarkable mechanical properties, and the pronounced tendency of cells to dedifferentiate when cultured [21]. Cellular scaffolds play a critical role in CTE by enabling 3D cultivation and stimulating cartilage formation and regeneration. These scaffolds must fulfill several criteria, including biocompatibility, suitable architecture, degradability, and optimal biological and physicochemical properties while supporting the cartilage phenotype [22]. The design of an ideal scaffold involves selecting suitable biomaterials and techniques to establish a reproducible framework that supports cell adhesion, growth, and migration while promoting cartilage-specific ECM production and maintaining cell morphology [2].

Scaffolds in CTE must be fabricated from biocompatible and biodegradable materials, with manufacturing techniques that allow precise control over shape, size, and microstructure. Polymeric scaffolds, particularly hydrogels, have garnered considerable attention because of their versatility and ability to adapt [23]. Combining hydrogels with 3D and 4D printing or rapid prototyping enables the automated and precise creation of intricate 3D structures with customizable properties. This approach uses biocompatible materials to control architecture, degradation, and mechanics. Bioprinting advances this further by incorporating living cells (bioink), boosting the potential for tissue regeneration and clinical use [24]. In addition, the success of CTE relies heavily on scaffold materials that support cell functions [25]. Traditional materials offer passive support with static properties and limited bioactivity, while smart materials are dynamic, responsive to stimuli, and highly bioactive, mimicking natural tissues. On the other hand, smart materials actively interact with their environment, responding to stimuli such as temperature and pH while exhibiting adaptable mechanical properties [26]. Their high bioactivity enables them to mimic natural tissues, making them ideal for use in CTE or drug delivery applications [27]. The comparison of traditional and smart materials in tissue engineering is highlighted in Table 1. In CTE, smart materials provide innovative and versatile solutions, overcoming the limitations of conventional materials by providing dynamic, responsive, and biomimetic scaffolds [28]. Though smart materials enable advanced tissue engineering and drug delivery, they are more complex and require sophisticated fabrication techniques [29].

## 4. The Selection of Materials in Cartilage Tissue Engineering

The effectiveness of tissue engineering primarily relies on selecting materials, which form the foundation for scaffold creation to support cell attachment, growth, proliferation, and differentiation [32]. The choice of materials for biomedical applications has evolved significantly, focusing on developing advanced, intelligent materials to meet specific tissue engineering needs [33]. One of the most important aspects of CTE is the development of scaffolds that support chondrogenesis and preserve the chondrocyte phenotype to promote cartilage regeneration. Traditionally, various natural or synthetic materials have been used in CTE. However, the widespread clinical application of synthetic polymers is hindered by several disadvantages, including limited biocompatibility, which may trigger inflammatory responses, poor cell adhesion due to the absence of bioactive sites, lack of bioactivity, and a hydrophobic nature that can restrict nutrient exchange and cell infiltration. Additionally, degradation issues, such as unsynchronized breakdown with cartilage regeneration or the release of toxic byproducts, further limit their effectiveness. Moreover, high costs, scalability challenges, and complex processing requirements pose significant barriers to their practical use in CTE [34,35].

**Figure 3:**
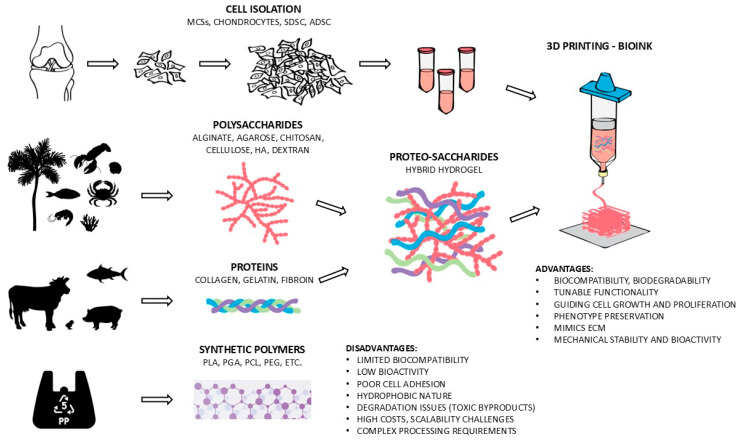
A schematic overview of the various materials used in CTE, including natural materials (proteo-saccharide combinations) and synthetic materials [2].

Natural biopolymers, including polysaccharide-based (e.g., chitosan, hyaluronic acid (HA), alginate, etc.) and protein-based (e.g., gelatin, collagen) hydrogels, exhibit excellent biocompatibility, low immunogenicity, and ECM-mimicking properties, making them ideal for CTE [2]. Polysaccharides stand out due to their biodegradability, tunable functionality, and structural similarity to cartilage ECM, positioning them as highly favorable materials for scaffold development in CTE. For instance, gellan gum (GG), a polysaccharide-based material, has been widely studied due to its ECM-mimicking properties relevant to articular cartilage [36,37]. In addition, proteins have emerged as exceptional candidates for smart materials due to their programmable self-assembly and ability to mimic cartilage ECM [38]. Moreover, proteins, such as collagen, enzymes, and antibodies, are vital in maintaining cell structure and function. Their unique properties, including complex 3D structures and specific biological functions, make them ideal building blocks for smart materials in CTE. For instance, collagen, a major component of cartilage ECM, can be used to create biomimetic scaffolds that promote cell adhesion, proliferation, and differentiation [39,40]. Incorporating proteins into smart materials enhances biocompatibility, strengthens mechanical properties, and enables dynamic responsiveness to physiological stimuli, advancing cartilage repair and regeneration [41]. Further improvements can be achieved by integrating polysaccharides with proteins, forming innovative “proteo-saccharide” formulations that enhance physiological signaling and mechanical resilience [2]. Figure 3 represents a schematic overview of the various materials used in CTE, including natural materials (proteo-saccharide combinations) and synthetic materials [2].

## 5. Smart Hydrogels in Cartilage Tissue Engineering

Hydrogels have transformed from passive, water-absorbing networks to dynamic, stimuli-responsive systems capable of mimicking biological intelligence. This transition involves strategic modifications to their chemical composition, physical architecture, and functional design, enabling them to interact with complex biological environments in real time [42].

Unlike traditional hydrogels, which remain static after synthesis, SHs have lately received much attention for their ability to undergo physical or chemical transformations in response to external stimuli or slight environmental changes [42,43]. Stimuli-responsive hydrogels (SRHs), in particular, are among the most interesting SHs due to their unique ability to change properties in response to physical (e.g., temperature, light, electromagnetic fields, pressure, and ultrasound), chemical (e.g., glucose, pH, and ionic strength), and biological (e.g., specific molecules and enzymes) stimuli [44], thereby enabling controlled cell delivery or drug release [45]. Unlike static hydrogels, SHs are characterized by dynamic covalent bonds and non-covalent interactions [46]. Moreover, SRHs feature essential characteristics to mimic chondral ECM, such as shape memory (tunable sol-gel transition) and self-healing potential, customizable swelling behavior, excellent permeability, and mechanical strength, as well as controlled biodegradability synchronized with neo-cartilage formation [47,48]. Also, they allow minimally invasive application in liquid form, followed by a controlled spatiotemporal transformation into gel form [49]. Due to their responsive characteristics, they actively support tissue regeneration [29]. This adaptive behavior has revolutionized CTE by dynamically interacting with cells and adjacent microenvironments, thus providing tailored support for cartilage regeneration and repair [14].

However, optimal material selection with ideal characteristics and features is the most critical aspect of successful scaffold formulation in CTE [1]. Its success mostly depends on countless studies, mainly analyzing finished products post-synthesis. Typically, methods for assessing SHs involve a “trial and error” approach, which includes macroscopic and microscopic observations, rheological measurements, material bonding conformation, and hydrogel formation [50]. Moreover, researchers are eager to compose their groundbreaking scaffold to meet complex requirements in CTE, thus dictating its rapid advancement by exploring new physical and chemical stimuli and multi-stimuli responsive hydrogels [23,51]. The following parts of the article describe novelties and important subheadings of smart polymers, fabrication techniques, unique features, and innovative design approaches of SHs in CTE.

### 5.1. Stimuli-Responsive Hydrogels

#### 5.1.1. Thermoresponsive Hydrogels

**Figure 4:**
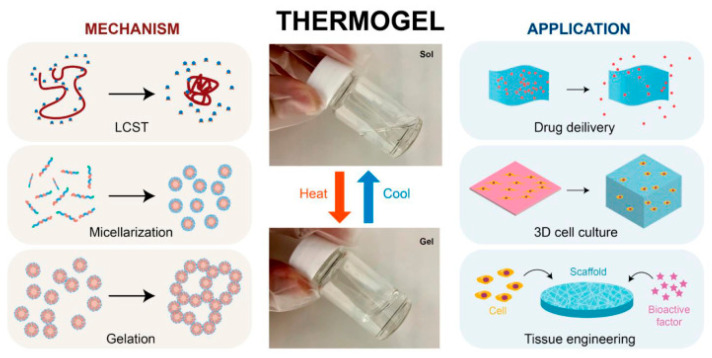
A representative illustration of thermogelling activity highlights various uses of thermogels and their thermoresponsive mechanisms [55].

Temperature-sensitive hydrogels are among the most common SHs in the literature [52]. These hydrogels demonstrate changes in volume, swelling dynamics, and permeability regarding differences in ambient temperature. They consist of hydrophilic and hydrophobic units at the molecular level, which undergo a gel-sol transition phase based on their lower or upper critical transition temperature (LCST and UCST, respectively) [30,53]. These materials react to temperature changes at body temperature, making them ideal for injectable hydrogels [54,55]. As a result, thermosensitive hydrogels demonstrate increased swelling capacity and expansive characteristics (Figure 4) [56].

Thermoresponsive hydrogels are biocompatible materials that can transition from a polymer state to a gel form at physiological temperatures. Typical examples are chitosan, poly (ethylene glycol)-poly (acrylic acid) block copolymers, and poly(N-isopropyl acrylamide) (pNIPAAm) [57]. The latter undergoes a gel-sol phase at 32 °C. Combined with chitosan, it contributes to the chondrogenic differentiation of mesenchymal stem cells (MSCs) [58]. In another similar study, Mellati et al. seeded thermo-sensitive chitosan-g-pNIPAAm hydrogels with MSCs. They demonstrated support for the differentiation into chondrocytes [59]. In vivo and in vitro studies have also shown that thermosensitive chitosan hydrogels effectively promote the adhesion of MSCs and increase cell volume [30]. Zhou et al. used transforming growth factor β1 (TGF-β1) enriched poly(ε-caprolactone)-poly(ethylene glycol)-poly(ε-caprolactone) (PCL-PEG-PCL) injectable and biodegradable hydrogel, which provided a thermoresponsive release of TGF-β1 in knee cartilage defects in rats [60]. They reported that a cell-free hydrogel enriched with growth factors has a high potential and can repair in vivo cartilage. Abbadessa et al. fabricated a thermo-responsive triblock copolymer of polyethylene glycol (PEG) and partially methacrylated poly[N-(2-hydroxypropyl) methacrylamide mono/dilactate]. Integrating polysaccharides mechanically stabilized the hydrogel and improved its printability [61].

#### 5.1.2. pH-Responsive Hydrogels

The pH-responsive hydrogels contain acidic or alkaline functional groups. Depending on the pH of the surrounding tissue, acidic groups deprotonate in alkaline environments and alkaline groups ionize in acidic environments. These changes involve hydrogen bonding between the polymer chains, electrostatic interactions, and the ion concentration inside and outside the hydrogel, which results in a swelling or shrinking of the hydrogel [30]. Acidic pH-responsive hydrogels that consist of alkaline groups [62] are synthesized from monomers like N, N-dimethylaminoethyl methacrylate (DMAEMA), N, N-diethylaminoethyl methacrylate (DEAEMA), and acrylamide (Aam). On the other hand, alkaline pH-responsive hydrogels consist of acidic groups [63] and are primarily synthesized from monomers like acrylic acid (AAc) and methacrylic acid (MAAc) [30]. A pH-responsive chondroitin sulfate—PEG adhesive hydrogel was developed by Strehin et al. Changes in the precursor solutions’ initial pH were discovered to influence the stiffness, swelling properties, and gelation kinetics. Higher pH reduced gelation time, leading to fewer amide bonds and a softer hydrogel. The hydrogel’s adhesion strength was ten times stronger at neutral pH than that of fibrin glue. Also, embedded cells remained viable and metabolically active after the transition [64]. Liang et al. fabricated dual-sensitive SHs based on chitosan and carrageenan, responsive to pH and ionic strength. It enhanced the chondrogenic differentiation of ATDC5 cells ex vivo [65]. Sá-Lima et al. created pH-sensitive hydrogels from chitosan-β-glycerophosphate-starch that can promote chondrogenic differentiation of adipose-derived stromal cells (ADSCs) [66]. In another study, a biphenyl-tripeptide supramolecular hydrogel was developed as a biomimetic ECM scaffold, responding to temperature, ion induction, and pH changes, with its gelation behavior influenced by amino acid arrangement. The tested formulations promoted chondrocyte proliferation, ECM secretion, and hyaline cartilage formation, demonstrating the significant potential for CTE [67]. However, studies on pH-responsive hydrogels in CTE have mostly been limited to in-vitro experiments [68].

#### 5.1.3. Hydrogels Responsive to Magnetic Fields

Magnetically sensitive hydrogels have recently been introduced into biomedical research to increase the biological activities of cells, tissues, or organs. The core parts of magnetic hydrogels are a matrix hydrogel and a magnetic element that has been added to the matrix [69]. They can be activated or adjusted without physical contact, thus precisely reacting to external magnetic fields and regulating the cellular environment’s physical, biochemical, and mechanical properties [70]. Specifically, these hydrogels display enhanced mechanical properties, allow magnetically guided bioactive molecule delivery, and enable non-invasive hydrogel remodulation through external magnetic fields [71,72]. Since electromagnetic stimulation has shown great potential to promote osteogenic differentiation of MSCs, it has lately become an interesting research field in CTE [69]. Recently, iron oxide-based superparamagnetic and biocompatible magnetic nanoparticles (MNPs) have become more widely used to create magnetically sensitive hydrogels for tissue engineering [69]. Zhang et al., in their study, mixed polyvinyl alcohol (PVA) modified iron oxide (Fe_3_O_4_) magnetic nanoparticles (MNPs) with a hybrid hydrogel made of HA, collagen type II (COL II), and PEG. It promoted the behavior of BMSCs in vitro and had comparable microstructure and chemical components to native hyaline cartilage [73]. Similarly, Huang et al., in their study, fabricated magnetic nano-composite hydrogel made of PVA, COL II, and Fe_3_O_4_. The hydrogels exhibited various promising compressive performances at different ratios and provided a new approach to cartilage defect treatment [74]. In a study by Huang et al., osteoconductive and osteoinductive superparamagnetic Fe_3_O_4_ nanoparticles and hydroxyapatite nanoparticles were incorporated into a di-block copolymer-based thermo-responsive injectable hydrogel made of methoxy(PEG)-polyalanine. Incorporated nanoparticles increased the complex viscosity and decreased the gelation temperature. Also, nanoparticles modulated bio-markers of bone differentiation and mineralization [75]. In another study, ADSCs were cultivated in a hyaluronan microenvironment, followed by a pulsed electromagnetic field treatment (PEMF). During chondrogenic induction, PEMF stimulation enhanced the expression of key chondrogenic genes such as aggrecan (ACAN), COL II, and SOX-9 [76]. Yang et al., in their study, merged kartogenin (KGN), a small molecule with the potential to induce BMSCs differentiation into chondrocytes, with the MNPs. The compound was then combined with dextran hydrogel and cellulose nanocrystals. KGN-MNPs labeled hydrogel displayed a prolonged, sustained release of attracted host cells and encouraged chondrogenic differentiation of BMSCs, resulting in increased in situ cartilage healing [77].

#### 5.1.4. Ion-Responsive Hydrogels

Hydrogels that respond to changes in ionic strength and transform from a solution to a gel form are commonly described in the literature [30]. Incorporating specific ions, such as Ca^2+^ or Mg^2+^, into polymer networks facilitates crosslinking and gel formation. This property is particularly useful for targeting inflamed tissues, where disrupted homeostasis often alters the ionic environment. Interestingly, nitric oxide (NO)—a gaseous signaling molecule elevated in inflamed joints—can indirectly influence local ionic conditions by modulating inflammatory mediators and cellular ion channels. In conditions like arthritis, excessive NO production leads to ionic imbalance, which can trigger ion-responsive hydrogels. Moreover, advanced hydrogel systems have been developed that combine ion responsiveness for gelation with NO-responsiveness for therapeutic action. For instance, an acrylamide-based hydrogel demonstrated effective arthritis treatment in mice by exploiting the altered joint microenvironment, including elevated NO levels to release anti-inflammatory agents in a controlled manner [78]. Such dual-responsive systems offer a promising strategy for on-demand drug delivery in inflammation-associated diseases [79].

Other ions have also been harnessed to engineer responsive hydrogels tailored to specific biological environments. Among these, calcium-sensitive hydrogels—particularly those based on alginate—have gained considerable attention. Alginate is a widely used, biocompatible polysaccharide that undergoes rapid gelation in the presence of divalent cations such as Ca^2+^. This makes it especially suitable for injectable or in situ forming systems that respond to calcium-rich environments, such as damaged or inflamed tissues. For example, an alginate/hyaluronic acid hydrogel system demonstrated the ability to support chondrogenic differentiation of ATDC5 cells and preserve their phenotype under calcium-stimulated conditions [80]. When combined with dual-responsiveness principles, such as those triggered by both ionic shifts and elevated NO levels in inflamed joints, such materials can be further optimized to enhance tissue-specific drug delivery and regenerative outcomes.

#### 5.1.5. Electrically Responsive Hydrogels

**Figure 5:**
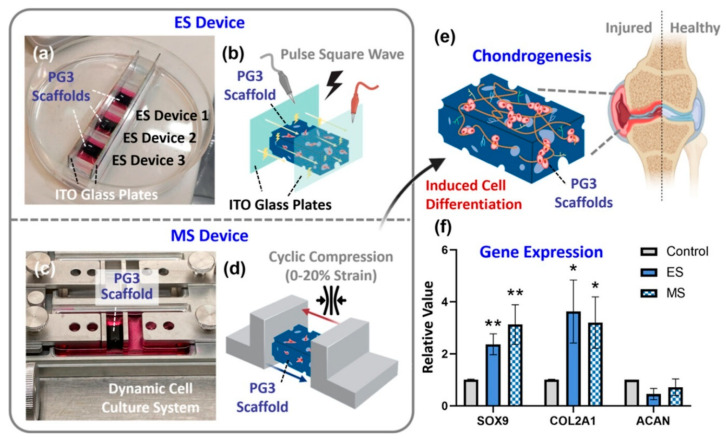
Schematic presentation of electrical and mechanical stimulation and their beneficial effect on chondrogenesis. Significant increases in chondrogenic gene markers SOX9 and COL2A1 were noted compared to the control group. ** *p* < 0.01, * *p* < 0.05. Reprinted with permission [82]. Copyright 2023. American Chemical Society.

Electrically responsive or conductive hydrogels are hydrogel-charged molecular networks. When surrounded by electrolyte solution and subjected to an electric field, ions reversibly move towards the corresponding positive or negative electrode [30]. The result is an uneven ion concentration distribution inside and outside the hydrogel, influencing osmotic pressure and ultimately causing macroscopic volume changes such as swelling, contraction, or shape deformation [81]. In other words, electrically responsive hydrogels convert electrical energy into mechanical energy [30]. Electrical stimulation promotes MSCs’ chondrogenesis, while mechanical stimulation enhances MSCs’ chondrogenic differentiation (Figure 5) [82]. Farooqi et al. utilized hydrogels as ECM matrices and applied electrical stimulation to induce proliferation, differentiation, and cell growth at sites of tissue defects. The study revealed the beneficial effects of both direct and indirect electrical stimulation [83]. In another study, researchers created cryogels using PVA and polyacrylic acid (PAA), comparing their behavior to chemically crosslinked gels. Unlike the chemically crosslinked gels, the physical cryogels exhibited increased elastic modulus with charge activation and swelling, attributed to the electrostatic stiffening of polymer chains at high charge densities. These responsive cryogels can fabricate biomimetic scaffolds for CTE and repair [84]. In another study by Zhang et al., the authors fabricated PVA hydrogel combined with sodium phytate (PANa), which exhibited conductivity and superb mechanical properties [85]. The hydrogel resisted tensile strain (over 600% with a tensile strength over 7 MPa) and compressive strain (up to 16 MPa when loading a strain of 90%). Moreover, the hydrogel presented promising anti-swelling properties, with a 50% increase in weight in 7 days and no significant swelling after 18 days, suggesting strong mechanical stability [85].

#### 5.1.6. Hydrogels Responsive to Piezoelectric Potential

Piezoelectric materials generate electrical charges in response to mechanical loading, promoting cell proliferation and differentiation, particularly in electrically responsive tissues such as bones and nerves [81,86]. Recent studies have also investigated the integration of electrical stimulation in CTE, focusing on materials with conductive properties like polyvinylidene fluoride (PVDF) and graphene oxide. These hydrogels promise to mimic biomechanical-electrical transduction, which may play a role in joint homeostasis and cartilage repair. When subjected to dynamic mechanical forces, they autonomously generate an electrical potential in situ, increasing chondrocyte activity, promoting cell alignment, and stimulating cartilage matrix synthesis [87,88]. Among natural piezoelectric materials, COL and chitosan can be utilized directly or combined with synthetic polymers to produce bioinspired electroactive scaffolds [89,90]. Furthermore, hydrogels responsive to piezoelectric potential could play an important role in preventing chondrocyte dedifferentiation, a major challenge in CTE, in which low-level electrical stimulation, generated under physiological movement conditions, could maintain the chondrogenic phenotype, especially in long-term cultures or post-implantation period [91].

#### 5.1.7. Light-Responsive Hydrogels

Light-sensitive hydrogels have also been described in CTE. These hydrogels consist of a polymer network and photoreceptive components, i.e., molecules that absorb light and initiate structural or chemical transformation [92]. Among the photoreactive materials that produce light-responsive hydrogels, photocrosslinkable polymers are widely used in CTE. Methacrylated gelatin (GelMA), methacrylated hyaluronic acid (HAMA), and PEG diacrylate (PEGDA) have been described in the literature [93,94]. These enable light-triggered gelation and spatial cross-linking density control [94]. A photo-responsive hydrogel made of alginate-acrylamide hybrid gels and ferric ions has been created by Giammanco et al. ATDC5 cells cultivated on the hydrogel and irradiated for 90 min produced more than twice as much sulfated glycosaminoglycans (GAG) as dark controls, suggesting light stimulation can increase ECM production and chondrogenic activity [95]. The physicochemical characteristics of these hydrogels could be fine-tuned in a wavelength-specific manner to allow deep tissue penetration with near-infrared light or localized activation by exposing them to visible or UV light [96,97].

#### 5.1.8. Positive Effects of Responsive Hydrogels in Cartilage Tissue Engineering

In summary, SRHs offer several advantages in CTE due to their ability to alter their properties in response to various environmental stimuli. These hydrogels enable the controlled release of therapeutic agents like growth factors, promoting chondrogenic differentiation and cartilage regeneration. Thermo-responsive hydrogels, for instance, allow for minimally invasive delivery by transforming from a liquid to a gel at body temperature and conforming to irregular cartilage defects. Additionally, these hydrogels create a three-dimensional microenvironment that mimics the ECM and supports cell adhesion, proliferation, and differentiation. Their tunable mechanical properties can adapt to dynamic joint loading, which is crucial for the regeneration of functional cartilage in load-bearing areas. Moreover, responsive hydrogels can degrade in response to specific stimuli, enabling scaffold remodeling and natural tissue integration without long-term material persistence. These features make SRHs highly promising for enhancing cartilage repair’s structural and functional outcomes [98].

### 5.2. Complemented Smart Hydrogels

#### 5.2.1. Nanocomposite Materials

Nanocomposite hydrogels have emerged as promising materials in CTE due to their ability to mimic the complex structure and function of native cartilage. Integrating nanoparticles into a hydrogel matrix improves mechanical characteristics, bioactivity, and cellular interactions, addressing the limitations of conventional hydrogels. For instance, including various micro- and nanoparticles into injectable hydrogels has improved scaffold performance, enabling a novel and attractive strategy for cartilage regeneration [99]. Among them, carbon-based (carbon nanotubes, graphene, nanodiamonds), polymeric (polymer nanoparticles, dendrimers, hyperbranched polyesters), metallic nanoparticles (gold and silver), and non-metallic nanoparticles have been shown to influence the hydrogel’s functionality [100,101,102,103]. Moreover, the incorporated nanoparticles play an important role in SRHs [101].

Recent advancements have focused on integrating advanced nanoparticles into hydrogels to further enhance their functionality in CTE. These nanocomposite hydrogels may effectively resemble native cartilage components due to their high histocompatibility, revealing unique biological impacts favorable to tissue regeneration [104]. Including carbon-based nanoparticles greatly enhanced the mechanical characteristics, surface roughness, and hydrogels’ lubricity, successfully replicating the natural lubrication of the synovial tissue in manufactured cartilage [105,106]. Furthermore, graphene nanoparticles have been applied as drug-delivery agents due to their promising biocompatibility and adsorption capacity [103,107].

Polymer nanoparticles in hydrogels enhance the 3D hydrogel network and support the sustained release of drugs. Growth factors-loaded gelatin in combination with a matrix containing β1-polycaprolactone (PCL)-PEG copolymer nanoparticles in vitro shows high cell adhesion, biocompatibility, and expression of cartilage-specific ECM genes like ACAN and COL II [108].

Gold’s conductivity and silver’s antibacterial characteristics have been introduced to nanocomposite hydrogels in various fields, such as conductive scaffolds, biosensors, and drug delivery systems [104]. The addition of TiO_2_ nanoparticles in chitosan hydrogel improved the biomechanical properties of the hydrogel and decelerated the degradation of the scaffold, thus facilitating the growth of seeded cells [109]. Despite promising in vitro results, further research on metal nanoparticle biocompatibility to explore cellular behavior in vivo is mandatory.

#### 5.2.2. Bioactive Supplements

**Figure 6:**
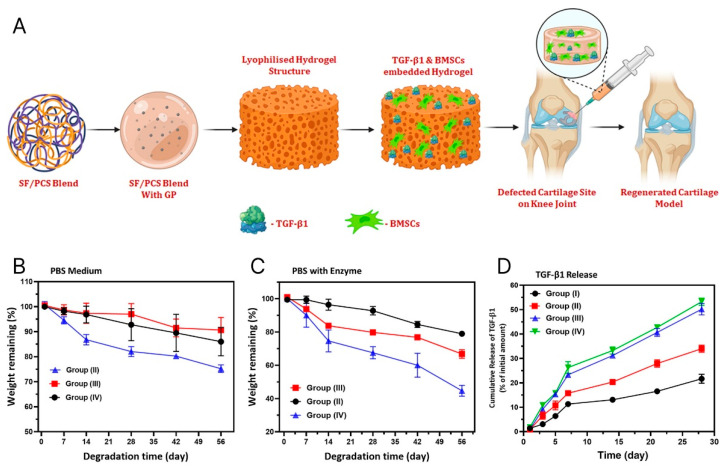
(**A**) Illustration of TGF-β1 and BMSCs encapsulated SF/PCS injectable hydrogel. (**B**) and (**C**) show in vitro biodegradation analysis (%) of blended hydrogel groups in PBS medium without and with enzymes (protease XIV and Lysozyme), respectively. (**D**) The diagram shows the in-vitro release of TGF-β1 from hydrogels of variable blending ratios of SF/PCS groups [112]. Copyright 2022, John Wiley & Sons.

Growth factors and other stimulants are essential molecules in native cartilage tissue. They serve as cell baits, induce cell responses, promote differentiation of mesenchymal stem cells into chondrocytes, promote the adhesion of chondrocytes to defects, and aid in the secretion of ECM. An innovative approach to enhance cellular response and tissue regeneration involves encapsulating biologically active substances or drugs within various particles and microspheres, which are then integrated into injectable hydrogels [110]. Moreover, growth factor-loaded SRHs possess high potential and benefits for acellular CTE [60]. For example, the TGF-β family of growth factors has been included in numerous studies [111]. Zheng et al. recently integrated BMSCs and TGF-β1 into the macromolecular silk fibroin blended with polylysine-modified chitosan polymer (SF/PCS), gelated with glycerophosphate Figure 6 [112]. The latter contributed to thermo-sensitive sustained release of growth factors, effectively regulating cartilage-specific gene expression like ACAN, COL II, and SOX-9 in vitro and in vivo (Figure 6). Notably, the expression levels of inflammation-related factors (IL-1β and IL-6) were considerably down-regulated compared to the control group [112]. Also, Park et al. in their study examined oligo(PEG) fumarate) and PEG-based injectable hydrogel loaded with gelatin microparticles of TGF-β1 and MSCs. They observed significant enhancement of ACAN and COL II expression [113].

Another promising delivery agent is nanostrontium ranelate. Through ionic stimulation of chondrocyte anabolism and insulin growth factor (IGF-1) activity, the drug enhances the production of cartilage matrix and, therefore, proteoglycan synthesis. Incorporated in chitosan-alginate-fibrin hydrogels, it showed a significant increase in proteoglycan production compared to hydrogel alone [114].

#### 5.2.3. Exosome-Loaded Hydrogels

Exosomes are nano-sized extracellular vesicles that mediate paracrine intercellular communication and transmission of bioactive cargo [115]. They have a significant function in both physiological and pathophysiological processes. Researchers have demonstrated their ability to promote tissue repair and their advantageous role in cartilage tissue repair [116,117,118]. Auspicious results were achieved in the field of targeted drug delivery [119,120]. By in situ crosslinking Pluronic F-127 and hyaluronic acid, Sang et al. created an injectable, thermosensitive hydrogel that could encapsulate and release primary chondrocyte-derived exosomes over a sustained duration. The hydrogel demonstrated a prolonged release of exosomes, effectively caused polarization of M1 to M2 macrophages, and positively controlled chondrocyte proliferation, migration, and differentiation. By encouraging the synthesis of the cartilage matrix, intraarticular injection of this exosome-incorporated hydrogel dramatically reduced the rate of cartilage degradation [121]. Liu created an exosome scaffold to prepare an acellular tissue patch (EHG) for cartilage regeneration using a light-induced imine crosslinking hydrogel glue that demonstrated exceptional operation ability, biocompatibility, and cartilage integration. In vitro, it had a favorable effect on hBMSCs and chondrocytes. Additionally, it increased cell deposition at cartilage defect locations and effectively merged with the native cartilage matrix, thus facilitating cartilage defect healing [122]. What is more, the combined effect of co-encapsulation of hMSCs and extracellular vesicles in a hyaluronic-acid-based hydrogel on cartilage regeneration showed enhanced potential to regenerate cartilage tissue, compared to hMSCs hydrogel alone [123].

#### 5.2.4. Positive Effects of Complemented Smart Hydrogels in Cartilage Tissue Engineering

In summary, complemented SHs significantly enhance the therapeutic potential of SHs in CTE by improving scaffold functionality, directing cellular behavior, and promoting tissue regeneration. Nanocomposite materials that include carbon-based (e.g., graphene, carbon nanotubes), polymeric, metallic (e.g., gold, silver, titanium dioxide), and non-metallic nanoparticles into hydrogel matrices contribute to improved mechanical strength, surface roughness, and lubrication features essential for mimicking native cartilage. These materials also support cell adhesion, viability, and chondrogenic differentiation. In addition, polymeric nanoparticles facilitate sustained drug and growth factor release, further enhancing cellular responses. Bioactive supplements such as growth factors, particularly members of the TGF-β family, have been successfully incorporated into hydrogels to stimulate MSCs differentiation into chondrocytes, upregulate cartilage-specific gene expression (e.g., ACAN, COL II, SOX-9), and attenuate inflammation. Moreover, integrating exosomes into injectable, thermosensitive hydrogels has demonstrated prolonged bioactive release, macrophage polarization from M1 to M2 phenotypes, and enhanced chondrocyte proliferation and migration. These cell-free systems also improve integration with native cartilage and promote ECM deposition. Collectively, these contribute to developing advanced, bioactive scaffolds capable of supporting robust and sustained cartilage regeneration [103,121,124].

## 6. Preferred Features of Smart Hydrogels in Cartilage Tissue Engineering

### 6.1. Injectability

**Figure 7:**
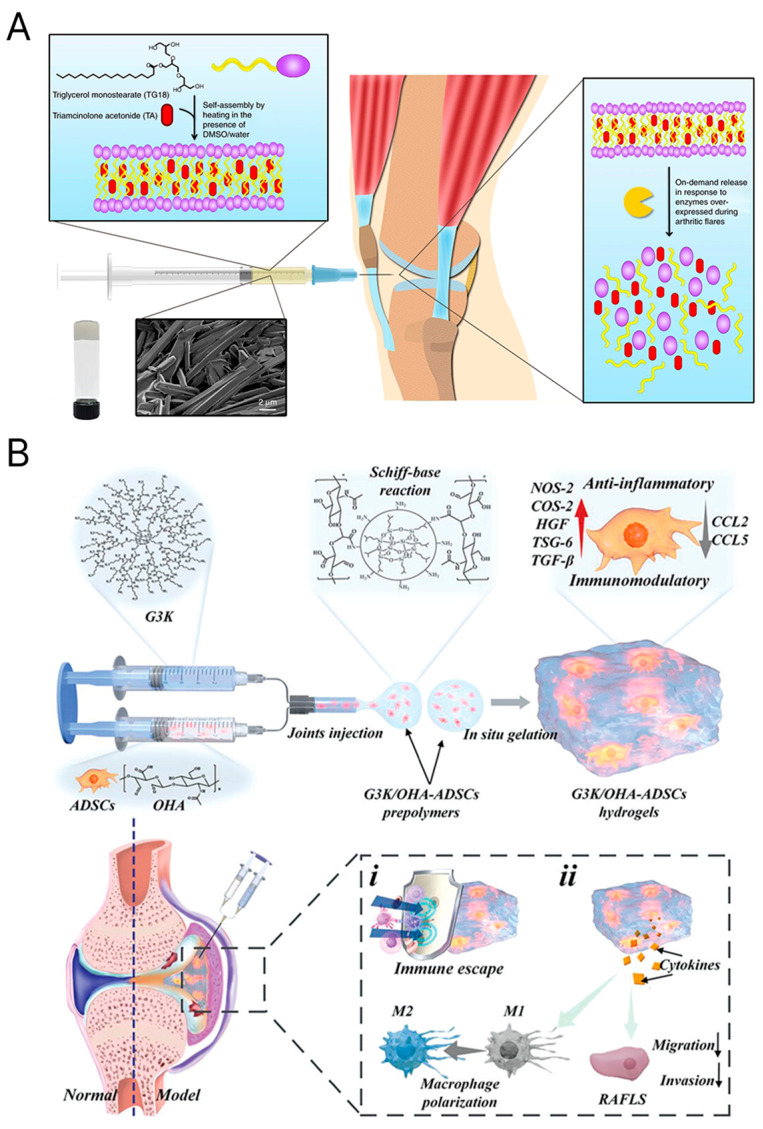
(**A**) A self-assembling injectable hydrogel with anti-inflammatory and immunomodulatory properties for the treatment of inflamed joints [54]. Copyright 2018, Springer Nature. (**B**) An innovative ECM-mimicking injectable hydrogel designed for adipose-derived stem cell encapsulation and delivery in RA treatment [54]. Copyright 2023, John Wiley & Sons.

SRHs allow minimally invasive in vivo administration, thus offering modern treatment options for biological applications in CTE without invasive surgical approaches [100]. Because of hydrogels’ characteristics, they may be injected into cartilage defects and gel in situ. This characteristic makes the environment conducive for cell proliferation and tissue regeneration. An injectable thermoresponsive hydrogel, for example, has been demonstrated to promote integration with surrounding tissue and facilitate chondrogenesis [125,126]. In another case, it has been shown that stimuli-responsive polymers, such as pNIPAAm, alter their characteristics in reaction to external influences like pH, light, or temperature, which makes them beneficial for cell sheet engineering and controlled drug delivery [127]. Moreover, hydrogels loaded with TGF-β3 have been shown to release the growth factor in response to temperature changes, significantly improving chondrocyte proliferation and cartilage repair [111]. Similarly, the bilayer porous scaffolds with GelMA hydrogels were developed via 3D printing. The upper layer was modified with bioactive peptides to adsorb TGF-β1 for cartilage repair, while the lower layer was blended with hydroxyapatite for subchondral regeneration. These scaffolds demonstrated promising therapeutic efficacy in vitro and in vivo, including restoring animal gait behavior [128]. ECM-inspired injectable hydrogel was recently introduced with encapsulated ADSCs for rheumatoid arthritis treatment. Figure 7 represents an injectable self-assembly hydrogel with anti-inflammatory and immunomodulatory properties for RA therapy [54,129].

### 6.2. Controlled Degradation

One of the key features of smart scaffolds is their ability to degrade under controlled conditions in response to exact environmental stimuli like changes in temperature fluctuations, pH, or enzymatic activity [130]. This controlled degradation is critical to ensure that the scaffold’s breakdown coincides with the rate of new tissue formation, thereby maintaining structural support throughout the healing process and preventing early scaffold disintegration. For instance, pH-responsive hydrogels have been engineered to degrade specifically in the acidic microenvironment characteristic of damaged cartilage [131]. These hydrogels release chondrogenic factors that stimulate tissue regeneration and provide essential mechanical support during the repair process [42]. It was demonstrated that variations in the precursor solutions’ initial pH might affect the final hydrogel products’ stiffness, swelling characteristics, and gelation kinetics [64]. In another study, novel dynamic hydrogels were prepared from O-carboxymethyl chitosan and a water-soluble dynamer via crosslinking by imine bond formation using an environmentally friendly method. The resulting soft hydrogels exhibited high storage moduli, excellent pH-sensitive swelling properties, and porous interconnected morphology. These hydrogels presented outstanding self-healing properties, making them promising candidates for wound dressing, applications in drug delivery, and tissue engineering [132].

### 6.3. Shape Memory

Shape memory polymers (SMPs) have proven highly useful because they can revert to a predefined shape when exposed to specific stimuli, such as temperature changes, making them ideal for minimally invasive implantation. Another significant characteristic of the scaffold is its mechanical adaptability, enabling it to modify its properties in response to external forces like compression or shear stress. This dynamic support is essential for CTE as it replicates the load-bearing capacity of native cartilage. Thus, mechanoresponsive smart scaffolds have emerged as a promising approach in CTE, aiming to mimic the dynamic mechanical environment of native cartilage. These scaffolds are designed to respond to mechanical stimuli, promoting chondrogenic differentiation and ECM production [16,17]. Similarly, SMPs have been employed to adjust their shape in response to external stimuli, enabling minimally invasive implantation and enhanced integration with surrounding tissues. These advancements underscore the potential of mechanoresponsive smart scaffolds to enhance cartilage repair and regeneration outcomes [86,133]. In addition, utilizing advanced fabrication techniques like 3D printing enables the creation of scaffolds with precise architecture and tailored mechanical properties. For example, 3D-printed gelatin/hydroxyapatite scaffolds were developed to support the adhesion, proliferation, and growth of human umbilical cord blood-derived mesenchymal stem cells (hUCB-MSCs) and promote chondrogenic differentiation in vitro. Moreover, cartilage defects were successfully repaired in a pig model by implanting the scaffold containing hUCB-MSCs at the location of the articular cartilage lesion [134].

### 6.4. Controlled Drug and Growth Factor Delivery

A critical potential SRHs offer is sustained and controlled drug delivery [67,135,136,137]. In general, to control the release of bioactive chemicals and ensure the accommodation of viable cells for CTE, the optimum pore size and density in the hydrogel and well-paced biodegradability are crucial criteria [138]. Hydrogels significantly benefit drug delivery, particularly for hydrophobic substances and macromolecule cargos. Specifically, several SHs that respond to internal or external stimuli have been produced and intensively researched, demonstrating great potential in drug delivery while overcoming the disadvantages of standard drug carriers [139,140]. Chauhan et al., in their study, created SRHs by crosslinking oxidized pullulan and PEG. The hydrogel was then loaded with dexamethasone (DEX), providing continuous medication administration based on the pH [141]. Similarly, Wang et al. synthesized a pH-sensitive hydrogel formed of vinyl pyrrolidone, hydroxyethyl methacrylate, and MAAc phosphatidylcholine. Within the pH range of 6.0–7.4, this polymer provides a transition into a gel state. The study found that this hydrogel system can be an intelligent glucocorticoid carrier, exhibiting promising therapeutic effects in inflammatory conditions [142].

Lately, studies have shown the potential of magnetic hydrogels as a drug release and targeting system. A Magnetic response PVA hydrogel showed the remote ability to adjust wettability and protein adsorption regarding the intensity of the external magnetic field, indicating a broad spectrum of applications in tissue engineering, drug delivery, and biosensors [143].

The low toxicity and gelation suitability of pNIPAAm have made it a suitable carrier for delivering bioagents in vivo. Furthermore, combining it with other substances like PEG or HA does not impact its thermosensitive characteristics [144,145]. The pNiPAAm polymer can also be modified using RGD sequences, a common peptide motif responsible for cell adhesion in the ECM, to improve cellular adhesion [146]. An innovative injectable and thermoresponsive hybrid hydrogel made of pNIPAAm and layered double hydroxides (LDHs) was introduced in another work by Yang et al. to deliver small interfering RNA (siRNA) for the inhibition of glyceraldehyde-3-phosphate dehydrogenase (GAPDH). They reduced 80–95% of GAPDH enzyme activity compared to the control group [147].

In a study by Deng et al., TGF-β3 was directly loaded with hMSCs into poly-D, L-lactic acid/PEG/poly-D, L-lactic acid (PDLLA-PEG) hydrogel and PDLLA-PEG with the addition of HA, and cultured in vitro [148]. The construct exhibited controlled release of TGF-β3 without using extra TGF-β3 in the culture medium. Hydrogels for delivering anti-inflammatory cytokines, including IL-10, were demonstrated in another study [149]. Zhang et al. utilized a thermosensitive hydrogel based on poloxamers loaded with glucosamine. After intra-articular administration in rabbits with induced osteoarthritis, swelling and inflammatory factors decreased [150]. A HA-based self-assembling hydrogel for IL-10 sustained administration was created by Soranno et al. The results showed reduced local and systemic proinflammatory cascades in a mouse model of acute renal damage [151]. Even though the latter does not directly relate to the field of chondrocytes, the applications of such findings to osteoarthritis are up-and-coming.

### 6.5. Self-Healing Potential

Intelligent hydrogels having self-healing characteristics, or the capacity for a material to regain its original structure after injury through noncovalent interactions, dynamic covalent connections, and physical bonding, have recently been studied [152,153]. Self-healing materials for cartilage repair must preserve the mechanical characteristics of cartilage, minimize local friction with zwitterionic or anionic polymers and natural polysaccharides, and prevent further degeneration of injured cartilage [154]. Moreover, they may support the distribution of essential bioactive molecules involved in molecular signaling, like genes, growth factors, and small-molecule drugs [122,155].

In research by Yu et al., they presented a multifunctional, self-healing hydrogel made of adipic dihydrazide, HA, and furylamine (furan) [156]. The advantages of combining the Diels–Alder click reaction and acyl hydrazone bond, which preserved mechanical characteristics and self-healing properties, respectively, indicated enormous potential in CTE. Notably, the compressive modulus of the hydrogels after the healing process was identical to that of the initial hydrogel. In another study, Wang et al. presented an injectable, self-healing, and dual-responsive hydrogel based on hydrazide-modified PEG (PEG-DTP) and oxidized sodium alginate with potential in CTE [157]. PEG-DTP improved physical characteristics and showed a reversible sol-gel transition triggered by pH. Ultimately, it displayed around 100% self-healing capacity following a breakage. Jeong et al. introduced injectable hyaluronate hydrogels formed of adamantane-modified HA (HA-Ad) and β-cyclodextrin-modified HA (HA-CD) [158]. It demonstrated superior mechanical properties such as self-healing and shear thinning with high cell viability of encapsulated MSCs. Recently, Roh et al. fabricated a polysaccharide-based hydrogel using glycol chitosan, oxidized hyaluronate (OHA), and adipic acid dihydrazide reinforced with alginate [159]. The latter reinforced the self-healing potential and improved the biomechanical characteristics of the hydrogel. Also, it showed great potential for chondrogenic differentiation of ATDC5 cells in vitro.

Moreover, photoresponsive self-healing hydrogels incorporating acrylic groups have emerged as a promising group of smart biomaterials. These hydrogels allow in situ reversible crosslinking under specific light stimuli, benefiting CTE by providing dynamic mechanical stability and autonomous repair capabilities [160]. These hydrogels demonstrated repeatable self-healing potential and the ability to support chondrocyte function [161].

**Figure 8:**
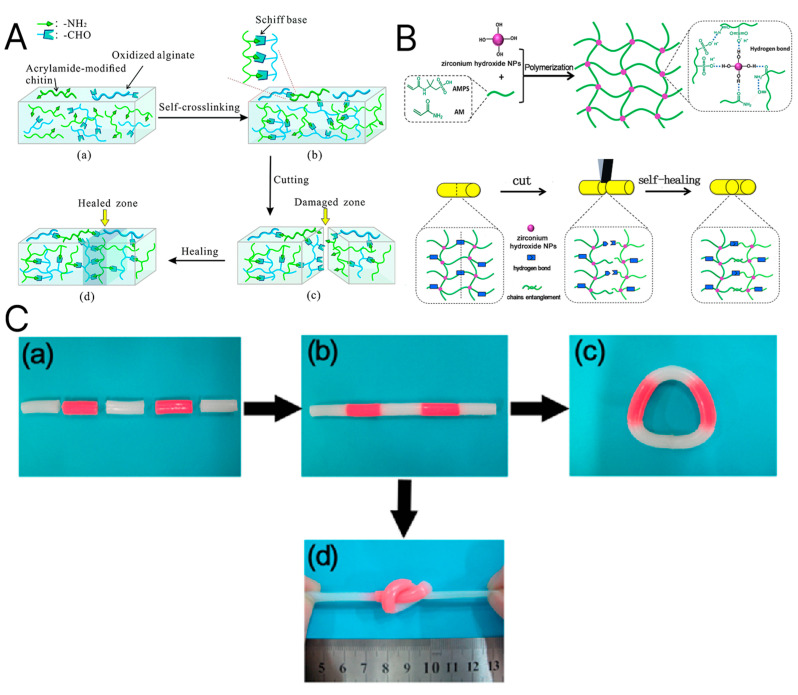
(**A**) Schematic illustration of the self-healing process. (**a**,**b**) Schiff-base linkage forms dynamic covalent bonds between aldehyde and amino groups. (**c**,**d**) If separated and put in contact, the hydrogel heals autonomously [166]. (**B**) A similar effect is noted in zirconium hydroxide nanocomposite hydrogels, forming hydrogen bonds. (**C**) The separated blocks were connected at room temperature without an external stimulus (**a**,**b**). The healed hydrogel showed mechanical stability, allowing it to be brought into a circle (**c**) as well as be knotted and stretched (**d**). Reprinted from Publication [167]. Volume 140. Copyright 2017, with permission from Elsevier.

Self-healing hydrogels have undergone tremendous studies to increase their mechanical strength, yet these systems continue to face significant difficulties in the dynamic and mechanically demanding environment [42]. To surmount the issue, hydrogels crosslinked by several dynamic and covalent bonds have recently been created [42,162,163]. Intriguingly, in a recent study, Ding et al. combined the networks of PVA hydrogel and acrylamide-modified chitosan crosslinked with oxidized alginate to create an interpenetrating polymer network (IPN) [164]. The mechanical strength was mainly improved by Schiff-base linkage (-HC=N-), e.g., dynamic covalent bonds between the amino groups (-NH_2_) and the aldehyde groups (-CHO), that enable the hydrogel to mend itself without external stimulation (Figure 8) [165,166]. Similarly, Jiang introduced a novel zirconium hydroxide crosslinking nanocomposite hydrogel that combines high toughness and self-healing efficiency (86%) at ambient conditions. The hydrogen-bonding network showed tunable characteristics regarding the molar ratio of zirconium hydroxide [167].

However, despite the latest advances in developing self-healing hydrogels that stimulate physical, chemical, and physiological repair of injured cartilage, mechanical strength seems to be the locus minor of the concept [154]. Thus, it continues to remain of utmost importance in CTE.

## 7. Future Outlooks into AI-Driven Hydrogel Design Through Omics-Based Approaches

**Figure 9:**
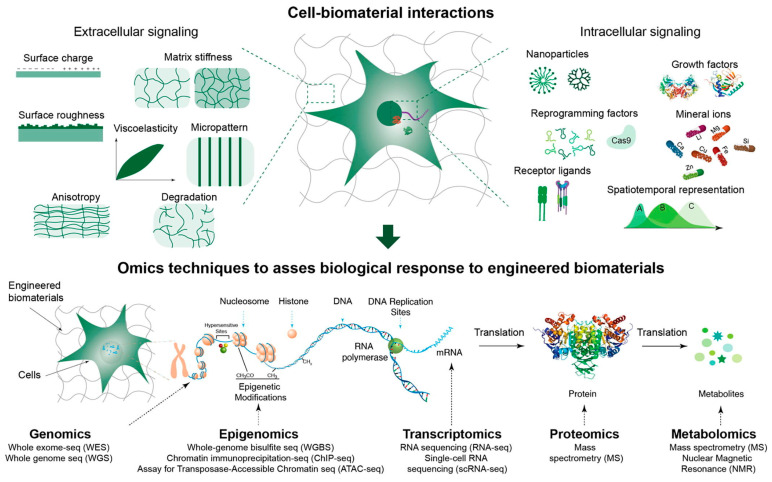
Biophysical and biochemical characteristics of selected biomaterials dictate interactions with cells. Omics techniques provide advanced insights into biological responses. Reprinted from Publication [169]. Volume 64. Copyright 2023, with permission from Elsevier.

Current state-of-the-art methods in hydrogel design and optimization often rely on repeated, time-consuming, and expensive experiments, which slow the advancement of hydrogel development. With the rapid growth of artificial intelligence (AI) and the increasing availability of material data, AI-driven design and optimization of hydrogels for biomedical applications have emerged as a promising bridge to revolutionary breakthroughs in materials science. AI offers advantages such as a complex composition-process-structure-property model for prediction and optimization, high-throughput screening, automated material discovery, and optimized experimental design [168]. Moreover, integrating omics technologies, including transcriptomics, proteomics, and spatial-omics, provides a novel analytical framework for evaluating the efficacy of SHs in CTE. Predicting the biological reaction to created biomaterials using omics-based methods has the potential to transform biomedical research and displace the conventional “trial and error” method in the future (Figure 9) [169]. Researchers aim to leverage the integration of AI-driven methodologies and omics-based analyses to develop hydrogels with tailored properties that align with specific cellular and tissue requirements. This integration facilitates the creation of more effective and personalized hydrogel-based therapies for cartilage repair and regeneration [168].

**Figure 10:**
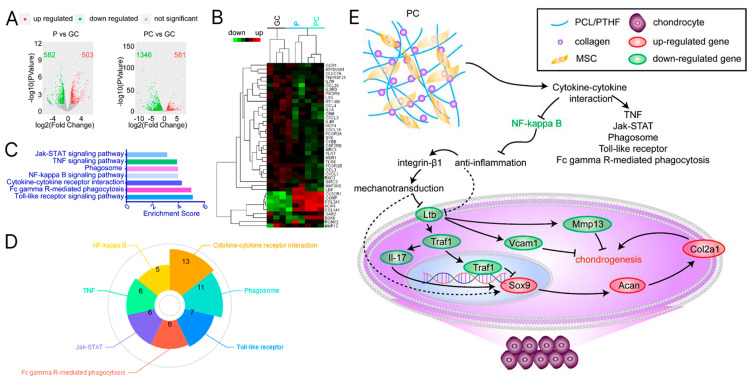
(**A**,**B**) Transcriptome profiles of chondrogenic differentiation of MSCs driven by nanofibers with (PC) and without collagen I (P). (**C**,**D**) KEGG pathway analysis predicts seven candidates for signaling pathways. (**E**) The proposed mechanism underlines chondrogenesis driven by PC nanofiber membranes. Reprinted from Publication [175]. Volume 178. Copyright 2018, with permission from Elsevier.

Innovations in DNA-functionalized bioinks illustrate the synergy between omics and materials science. By embedding aptamers or plasmid DNA into HA networks, these hydrogels not only respond to external stimuli but also upregulate chondrogenic markers (e.g., SOX9) through controlled gene delivery, as demonstrated by single-cell RNA-seq (scRNA-seq) [170]. Transcriptome profiling allows precise characterization of cellular responses to hydrogel microenvironments, as demonstrated by RNA-sequencing studies showing differential gene expression associated with osteogenic and chondrogenic differentiation in hBMSCs cultured on mechanically tuned hydrogels [171,172]. Thus, signaling pathways related to ECM remodeling, ion channel activity, and focal adhesion were upregulated in hydrogels with optimized stiffness and dynamic mechanical stimuli, highlighting their ability to mimic the biomechanics of native cartilage [171,173]. In a recent study, the interactions of nanoparticles with hMSCs were also investigated using RNA-seq. This showed that more than 4000 genes were regulated differently. The study provided transcriptomic insights into the role of nanomaterial-triggered surface-mediated cellular signaling and enabled the development of nanomaterial-based therapeutics for regenerative medicine [174]. Transcriptomics-based techniques can reveal specific essential signaling pathways involved in cartilage tissue regeneration from stem cells. Jiang et al. used transcriptomics analysis to determine the signaling pathways that MSCs cultivated on PCL/polytetrahydrofuran-urethane mixes with and without collagen I (COL I) used to undergo chondrogenic development (Figure 10). Unexpectedly, the findings validated a soft matrix devoid of COL I’s chondrogenic capability [175]. RNA-seq was also used to investigate the chondrogenic potential of silicon calcium phosphate (SiCP)-based bioceramic materials. Not only was it able to induce osteogenesis in MSCs, but also more than 450 genes related to chondrogenesis were upregulated [176]. Proteomic analyses confirm the hydrogel’s performance by quantifying the ECM’s cartilage-specific components, such as COL II and ACAN, which are critical for functional tissue regeneration [42,177]. A study using a proteomics-based method to examine de novo ECM proteins has demonstrated the vital role of chondroitin sulfate in promoting signaling pathways that regulate cartilage regeneration and chondrogenesis [178]. In another work, the osteochondral regeneration potential of a biphasic scaffold made from ECM from growth plates and articular cartilage was demonstrated using proteomics-based mass spectrometry. While over 330 proteins were discovered only in growth plate tissue, the authors isolated 32 exclusive to articular cartilage proteins. The specific proteins were later incorporated into a biphasic scaffold that promotes bone formation on one side and cartilage regeneration on the articular side [179]. Spatial-omics techniques, including near-infrared (NIR) imaging and spectroscopic methods, provide spatially resolved molecular insights into the integration of the hydrogel into the host tissue and allow detection of GAG retention and proteoglycan distribution—important indicators of successful cartilage repair [180,181]. In addition, scRNA-seq identified chondrocyte subpopulations in hydrogel scaffolds that correlate with enhanced deposition of ECM and reduced hypertrophy [180,182]. These -omics-guided approaches not only optimize hydrogel design by linking material properties to molecular outcomes but also ensure the controlled delivery of bioactive factors such as TGF-β1 and KGN that synergistically promote chondrogenesis while mitigating inflammatory responses [42,177,183]. By combining multi-omics data with functional assessments, researchers can systematically evaluate hydrogels’ biocompatibility, bioactivity, and long-term therapeutic potential and advance their translation into clinically viable strategies for cartilage regeneration [23,172,184].

## 8. Additional Considerations of Smart Materials in Cartilage Tissue Engineering

Although SHs have great potential in CTE, their clinical translation and commercialization remain limited due to significant regulatory, manufacturing, and cost-related challenges. Beyond translational hurdles, key material-related concerns, such as degradation behavior, potential toxicity, and long-term stability, must be addressed to ensure safety and efficacy in vivo. These factors represent critical barriers that must be overcome to fully realize the therapeutic potential of smart hydrogel systems in regenerative medicine.

### 8.1. Degradation, Potential Toxicity, and Long-Term Stability of Smart Hydrogels

The successful application of hydrogels in regenerative medicine hinges on a careful balance between degradation kinetics, biocompatibility, and structural stability. Hydrogel degradation must be timed to coincide with the rate of tissue regeneration or healing; however, achieving this synchronization remains a significant challenge due to the inherent complexity and variability of physiological environments. Hydrogels are typically degraded by hydrolytic or enzymatic pathways, with the degradation rate influenced by the polymer composition, degree of crosslinking, and environmental conditions such as pH, temperature, and ionic strength. PEG-based hydrogels, for example, can be developed for hydrolytic degradation, yet their by-products may vary in bioactivity and require thorough evaluation for safety and biofunctionality [185,186].

A critical concern arises when degradation occurs either too rapidly or too slowly. Rapid degradation may result in premature loss of mechanical support before tissue integration. At the same time, materials that are too durable may lead to fibrotic encapsulation or inhibit the formation of new tissue. This emphasizes the necessity of designing hydrogels with tunable degradation profiles tailored to specific clinical contexts [187].

Biocompatibility is another essential requirement for hydrogel-based biomaterials. Natural hydrogels derived from alginate, gelatin, or HA are generally well tolerated due to their biomimetic composition and low immunogenicity. On the other hand, synthetic hydrogels are advantageous due to their mechanical and structural versatility but may present cytotoxic risks. These risks often stem from residual unreacted monomers, degradation by-products, or reactive crosslinking agents [2,188,189]. To mitigate these issues, recent research has focused on developing bio-orthogonal crosslinking strategies and enzyme-responsive hydrogel systems that degrade into metabolically safe by-products such as lactic acid or simple sugars [190].

Regarding long-term stability, hydrogels must maintain their mechanical integrity and functionality over a clinically relevant period, particularly in load-bearing or slowly regenerating tissues. Numerous factors influence this stability, including hydrolytic degradation, mechanical fatigue, swelling-deswelling dynamics, and interactions with the host immune system. Hydrogels that remain structurally stable under laboratory conditions may degrade more rapidly in vivo due to enzymatic activity or oxidative stress in the tissue microenvironment [191]. Moreover, incorporating living cells or therapeutic agents into hydrogels can alter the internal physicochemical environment, accelerating degradation or modifying drug release kinetics. As a result, novel approaches such as nanocomposite reinforcement, microstructure optimization, and precision 3D bioprinting are being applied to increase mechanical robustness and regulate degradation behavior [192].

### 8.2. Challenges in Clinical Translation and Commercialization of Smart Hydrogels

SHs are emerging as a transformative class of biomaterials in regenerative medicine, drug delivery, and minimally invasive therapies. Their ability to respond to numerous physiological or chemical stimuli allows for on-demand behaviors such as controlled drug release, self-healing, and dynamic shape adaptability. Despite their potential, the clinical translation and commercial use of SHs face multiple interrelated challenges, particularly in regulatory approval, manufacturing scalability, and cost-effectiveness [193,194].

One of the most pressing issues in clinical implementation is the complexity of the regulations associated with smart hydrogel systems. These materials are often categorized as combination products, integrating drugs, biologics, and medical devices, making them subject to complex and rigorous evaluation by regulatory agencies such as the FDA and EMA [195,196]. The requirement for extensive in vivo testing, long-term biocompatibility testing, biodegradability assessments, and product-specific validation according to Good Manufacturing Practice (GMP) guidelines significantly extends the timeline and cost of development [197,198]. Furthermore, there is currently no standardized regulatory framework for stimuli-responsive materials, which leads to case-by-case testing that increases the unpredictability of approval pathways.

The scalability of production is another critical bottleneck. Many SHs are developed in laboratory-scale environments under strictly controlled conditions. Transitioning these formulations to industrial production involves significant challenges, including maintaining batch-to-batch reproducibility, functional responsiveness, and sterility without compromising the integrity of the hydrogel [199]. Sterilization techniques such as gamma irradiation, ethylene oxide, or autoclaving can degrade or deactivate functional components (e.g., thermosensitive or photoresponsive groups), thus requiring low temperature or aseptic processing workflows that add complexity and cost [192]. SHs incorporating biologics or cells add further complications, as these components are susceptible to processing conditions and require cold-chain logistics, specialized bioreactors, or encapsulation methods. This not only increases technical complexity but also regulatory oversight [200].

From a commercialization standpoint, cost-effectiveness is an important factor in clinical viability. The high cost of specialized polymers, complex synthesis procedures, sterile manufacturing environments, and regulatory compliance significantly raise the unit cost of smart hydrogel products [201]. Moreover, limited economies of scale and niche therapeutic applications can deter investment and reduce market penetration. To improve economic feasibility, efforts are underway to standardize hydrogel formulations, utilize modular design strategies, and develop ready-to-use platforms with simplified clinical applications [202].

Despite the existing challenges, significant progress has been made in commercializing smart hydrogel technologies. Injectable in situ-forming hydrogels have gained considerable attention in cartilage repair, with products like Cartistem^®^ (Seongnam-si, Republic of Korea) and GelrinC™ (Israel) reaching clinical trials and early market stages [203,204]. Thermo-responsive hydrogels, particularly those based on pNIPAAm, are now used in ocular drug delivery systems and as post-surgical anti-adhesion barriers, with several achieving regulatory approval in Asia and Europe [205,206]. In parallel, self-healing and 4D-printed hydrogels have emerged as cutting-edge biomaterials capable of reversible structural transformations in response to environmental stimuli, offering innovative applications in soft robotics, wound healing, and dynamic tissue models such as organ-on-chip devices [207]. Furthermore, expanding commercial hydrogel bioink platforms by companies like CELLINK, Allevi, and RegenHu has enabled SHs to integrate into advanced 3D bioprinting systems, supporting customizable, application-specific tissue engineering solutions [208].

## 9. Conclusions

Smart hydrogels have gained recognition as promising scaffolds for CTE due to their advantageous biomechanical and cell-friendly characteristics and active delivery of biomolecules, thus promoting chondral ECM deposition and ultimately closely mimicking native cartilage tissue. While advancements have been achieved, key challenges remain in achieving long-term stability, controlled degradation, and precise biomolecule delivery for effective in vivo applications. Modern design approaches have been shown to offer a pivotal role in precise step-by-step biomaterial selection. Undoubtedly, a mechanically defiant, firmly integrated hydrogel capable of autonomously responding to homeostatic disturbances through controlled drug release, self-regeneration, and maintenance of chondrocyte phenotype while stimulating chondrogenesis remains a primary objective. However, further studies, particularly those involving in vivo validation, are essential for clinical translation in the future.

## Figures and Tables

**Table 1:** The comparison of traditional and smart materials in CTE highlights their examples, advantages, limitations, and applications [23,27,28,29,30,31].

Category	Traditional Material Hydrogels	Smart Material Hydrogels
**Definition**	Inert, merely biocompatible, available, and easy to process.	Interact with the environment and respond to stimuli (e.g., temperature, pH).
**Examples**	**Natural hydrogels**: Collagen, fibrinogen, hyaluronic acid, alginate, chitosan.	**Stimuli-responsive hydrogels**: Crosslinked traditional natural and synthetic materials, pNIPAAm, DMAEMA, DEAEMA, AAc, MAAc.
	**Synthetic hydrogels**: PLA, PGA, PCL, PEG.	**Nanocomposites**: MNPs, Fe_3_O_4_, polypyrrole, polyaniline, graphene; carbon-based, polymeric, metallic, and non-metallic nanoparticles.
		**Bioactive supplements**: Growth factors, exosomes.
		**Shape memory hydrogels**: Polyurethane-based polymers.
		**Self-healing hydrogels**: Polymers with reversible bonds (e.g., hydrogen bonds, Schiff-base linkage).
**Advantages**	**Natural hydrogels**: Biocompatible, biodegradable, inherently bioactive.	**Stimuli-responsive hydrogels**: Controlled drug delivery, on-demand scaffold degradation.
	**Synthetic hydrogels**: Tunable mechanical properties, controlled degradation, reproducible synthesis.	**Nanocomposites**: Controlled drug release, enhanced mechanical properties.
		**Bioactive supplements**: Enhanced cell adhesion and differentiation.
		**Shape memory hydrogels**: Minimally invasive implantation, shape change in response to stimuli.
		**Self-healing hydrogels**: Self-repair, enhancing longevity.
**Limitations**	**Natural hydrogels**: Poor mechanical strength, batch-to-batch variability, rapid degradation.	**Stimuli-responsive hydrogels**: Complex fabrication and potential biocompatibility issues.
	**Synthetic hydrogels**: Lack of bioactivity, potential for inflammatory responses.	**Nanocomposites**: Potential toxicity of nanoparticles.
		**Bioactive supplements:** Short half-life, denaturation, burst release, diffusion limitations, and immune reactions.
		**Shape memory hydrogels**: Limited mechanical strength, slow response time.
		**Self-healing hydrogels**: Limited healing efficiency in some cases.
**Applications**	**Natural hydrogels**: Soft tissue engineering (e.g., skin, cartilage).	**Stimuli-responsive hydrogels**: Dynamic cell culture systems, drug delivery.
	**Synthetic hydrogels**: General tissue scaffolding, sutures, and implants.	**Nanocomposites**: Enhanced scaffolds for bone and CTE.
		**Bioactive supplements**: Enhanced cell adhesion and chondral differentiation, tissue-specific regeneration.
		**Shape memory hydrogels**: Minimally invasive implants.
		**Self-healing hydrogels**: Long-lasting scaffolds for tissue engineering and regeneration.

pNIPAAm—poly(N-isopropyl acrylamide), DMAEMA—N,N-dimethylaminoethyl methacrylate, DEAEMA—N,N-diethylaminoethyl methacrylate, AAc—acrylic acid, MAAc—methacrylic acid, PLA—polylactic acid, PGA—polyglycolic acid, PCL—polycaprolactone, PEG—polyethylene glycol, MNPs—magnetic nanoparticles.

## Data Availability

No new data were created or analyzed in this study. Data sharing does not apply to this article.
